# Test-time augmentation for deep learning-based cell segmentation on microscopy images

**DOI:** 10.1038/s41598-020-61808-3

**Published:** 2020-03-19

**Authors:** Nikita Moshkov, Botond Mathe, Attila Kertesz-Farkas, Reka Hollandi, Peter Horvath

**Affiliations:** 1 Biological Research Centre, Szeged, Hungary; 2 University of Szeged, Szeged, Hungary; 3 National Research University, Higher School of Economics, Moscow, Russia; 4 Institute for Molecular Medicine Finland, University of Helsinki, Helsinki, Finland

**Keywords:** Image processing, Machine learning

## Abstract

Recent advancements in deep learning have revolutionized the way microscopy images of cells are processed. Deep learning network architectures have a large number of parameters, thus, in order to reach high accuracy, they require a massive amount of annotated data. A common way of improving accuracy builds on the artificial increase of the training set by using different augmentation techniques. A less common way relies on test-time augmentation (TTA) which yields transformed versions of the image for prediction and the results are merged. In this paper we describe how we have incorporated the test-time argumentation prediction method into two major segmentation approaches utilized in the single-cell analysis of microscopy images. These approaches are semantic segmentation based on the U-Net, and instance segmentation based on the Mask R-CNN models. Our findings show that even if only simple test-time augmentations (such as rotation or flipping and proper merging methods) are applied, TTA can significantly improve prediction accuracy. We have utilized images of tissue and cell cultures from the Data Science Bowl (DSB) 2018 nuclei segmentation competition and other sources. Additionally, boosting the highest-scoring method of the DSB with TTA, we could further improve prediction accuracy, and our method has reached an ever-best score at the DSB.

## Introduction

Identifying objects at the single-cell level is the starting point of most microscopy-based quantitative cellular image analysis tasks. Precise segmentation of the cell’s nucleus is a major challenge here. Numerous approaches have been developed, including methods based on mathematical morphology^1^ or differential geometry^2,3^. More recently, deep learning has yielded a never-seen improvement of accuracy and robustness^4–6^. Remarkably, Kaggle’s Data Science Bowl 2018 (DSB)^7^ was dedicated to nuclei segmentation, and gave a great momentum to this field. Deep learning-based approaches have proved their effectiveness: practically all the teams used some type of a deep architecture in the first few hundred leaderboard positions. The most popular architectures included U-Net^4^, originally designed for medical image segmentation, and Mask R-CNN^8^, used for instance segmentation of natural objects.

Deep learning approaches for object segmentation require a large, and often pixel-wise annotated dataset for training. This task relies on high-quality samples and domain experts to accurately annotate images. Besides, analysing biological images is challenging because of their heterogeneity and, sometimes, poorer quality compared to natural images. In addition, ground truth masks might be imperfect due to the annotator-related bias, which introduces further uncertainty. Consequently, a plethora of annotated samples is required, making object segmentation a laborious process. One of the techniques utilized to improve the model is data augmentation^9^ of the training set. Conventionally, a transformation (i.e. rotation, flipping, noise addition, etc.) or a series of transformations are applied on the original images. Data augmentation has become the *de facto* technique in deep learning, especially in the case of heterogeneous or small datasets, to improve the accuracy of cell-based analysis.

**Figure 1:**
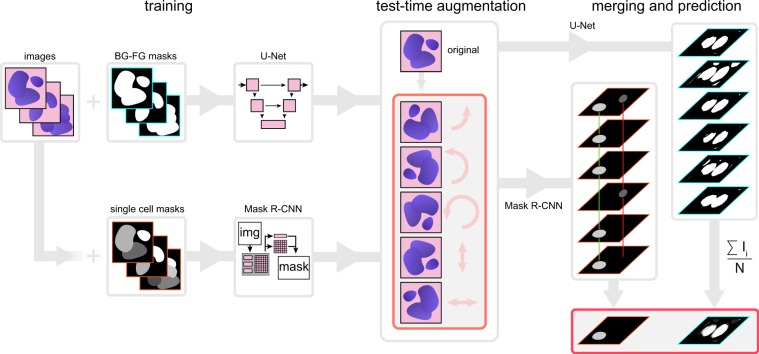
Principle of the proposed test-time augmentation techniques. Several augmented instances of the same test images are predicted, and the results are transformed back and merged. In the case of U-Net, pixel-wise majority voting was applied, while for Mask R-CNN a combination of object matching and majority voting was applied.

Another option of improving performance relies on augmenting both the training and the test datasets, then performing the prediction both on the original and on the augmented versions of the image, followed by merging the predictions. This approach is called ***test-time augmentation*** (Fig. 1). This technique was successfully used in image classification tasks^10^, for aleatoric uncertainty estimation^11^, as well as for the segmentation of MRI slices/MRI volumes^12^. A theoretical formulation^12^ of test-time augmentation has recently been described by Wang *et al*. Their experiments show that TTA helps to eliminate overconfident incorrect predictions. Additionally, a framework^13^ has also been proposed for quantifying the uncertainty of the deep neural network (DNN) model for diagnosing diabetic retinopathy based on test-time data augmentation. Its disadvantage is increased prediction time, as it is run not only on the original image, but on all of its augmentations as well.

In the current paper we assess the impact and describe cases of utilizing test-time augmentation for deep-learning models trained on microscopy datasets. We have trained deep learning models for semantic segmentation (when the network only distinguishes the foreground from the background, using the U-Net architecture) and instance segmentation (when the network assigns labels to separate objects, using the Mask R-CNN architecture) (Fig. 1). Test-time augmentation has outperformed single instance predictions at each test case, and could further improve the best result of the DSB, as demonstrated by the improvement of the score, changing from 0.633 to 0.644.

## Methods

### Dataset acquisition and description

We have used two datasets: fluorescent microscopy images (further referred to as ‘fluorescent’ dataset) and histopathology images (further referred to as ‘tissue’ dataset). Most of the images have come from the stage 1 train/test data of Data Science Bowl 2018. We also used additional sources^14–20^ and other data published in the discussion thread ‘Official External Data Thread’ (https://www.kaggle.com/c/data-science-bowl-2018/discussion/47572) related to DSB 2018. The images were labelled by experts using the annotation plugins of ImageJ/Fiji and Gimp. Both datasets were divided into three holdout train/test sets: approximately 5%, 15% (6 splits for each, cross-validation), and 30% (further referred to as ‘5’, ‘15’ and ‘30’ in the dataset name, respectively) of uncropped images were held out as the test set. The test sets (‘5’, first cross-validation split of ‘15’ and ‘30’) did not intersect.

**Figure 2:**
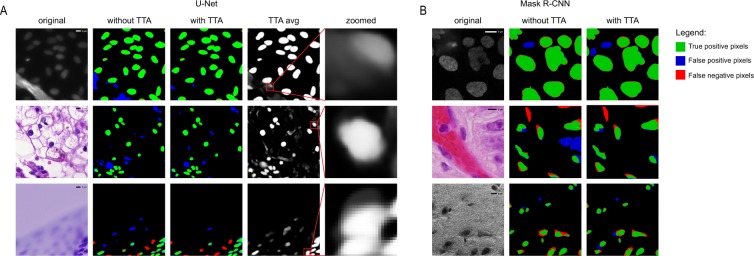
Examples of predictions. (**A**) U-Net predictions. First column - original image, second column - predictions without TTA compared to ground truth, third column - predictions with TTA compared to ground truth. Red indicates false negative pixels, green indicates true positive pixels and blue indicates false positive pixels. Dividing lines: yellow is false positive division of pixels into objects, and cyan is false negative division of pixels into objects. Fourth column - averaged TTA predictions before thresholding, fifth column - zoomed insets from the previous column. (**B**) Mask R-CNN predictions. Columns are the same as the first three columns in (**A**). Images in line 1 are examples of the fluorescent dataset, images in line 2 and 3 are examples of the tissue dataset.

We used the same augmentations (horizontal and vertical flip, 90°, 180° and 270° rotations) for training both architectures. The images were cropped to the size of 512 × 512 pixels. Crops from the same image were used only in either the train or test set. Images with a resolution of less than 512 × 512 were resized to that particular size. Sample images are shown in Fig. 2.

### Deep learning models and training

These augmented and cropped training data were used to train the models. For each dataset (5, 15 (6-fold cross validation) and 30 holdouts for both fluorescent and tissue images) separate models were trained. Additionally, we also trained U-Net without augmented data to analyse TTA performance on such a network as well (just 1 holdout 15 test set in that case).

Mask R-CNN (implementation^21^) is an extension of Faster R-CNN, the architecture for object detection. Solutions based on Mask R-CNN outperform the COCO 2016 challenge winners, and finished at the third place in Kaggle Data Science Bowl 2018^7^. The architecture of Mask R-CNN incorporates the following main stages: (1) Region proposal network (RPN) to propose candidate bounding boxes. It uses a backbone: a convolutional neural network which serves as a feature extractor. In this implementation it is possible to use *resnet50* or *resnet101* as a backbone, and we used *resnet101*. (2) Network head layers: they predict the class, box offset and an output binary mask for each region of interest (RoI). Masks are generated for each class without competition between the classes.

Following the strategy described by Hollandi *et al*.^5^, the network was trained for 3 epochs for different layer groups: first, all network layers were trained at a learning rate of 10^−3^, then training was restricted to ResNet stage 5 (ResNet consists of 5 stages, each with convolution and identity blocks including 3 convolutional layers per block) and head layers at a learning rate of 5 × 10^−4^, and finally only the head layers were trained at a learning rate of 10^−4^. The model was initialized with pre-trained weights (https://github.com/matterport/Mask_RCNN/releases/download/v1.0/mask_rcnn_coco.h5) on the COCO dataset. The loss function of the architecture was binary cross-entropy with ADAM^22^ (Adaptive Moment Estimation) solver, batch size 1, the number of iterations being equal to the train set size.

U-Net (implementation^23^) is an architecture originally designed to process biological images, which proved to be efficient, even when utilizing small training datasets. U-Net based solutions won the 2015 ISBI cell tracking challenge^4^ and Kaggle Data Science Bowl 2018. Its architecture consists of two main parts: (1) a down-sampling convolution network or encoder by which we obtain the feature representation of the input image, and (2) an up-sampling convolution network or decoder, which produces the segmentation from a feature representation of the input image.

We trained U-Net for 200 epochs at a constant learning rate of 3 × 10^−4^, and used a binary cross-entropy loss function with ADAM solver, batch size 1, the number of iterations being equal to the train set size.

Both U-Net and Mask R-CNN implementations are based on the deep learning framework Keras with Tensorflow backend. The training computations were conducted on a PC with NVIDIA Titan Xp GPU, 32 GB RAM and Core-i7 CPU.

### Test-time augmentation

Test-time augmentation includes four procedures: augmentation, prediction, dis-augmentation and merging. We first apply augmentations on the test image. These are the same as the augmentations previously applied on the training dataset. We predict on both the original and the augmented images, then we revert the transformation on the obtained predictions; this process is referred to as dis-augmentation. For example, when the prediction was performed on a flipped or rotated image, we restore the obtained prediction to its original orientation. The final merging step is not straightforward in case of Mask R-CNN, as the architecture is instance aware, thus the merging method has to handle instances. We have developed an extended merging method inspired by one of the DSB 2018 solutions^24^ (Fig. 1, right). For each detected object from the original image, we find the same detected objects in the augmented images by calculating intersection over union (IoU) between the masks. The minimum IoU threshold used to decide whether the objects found are the same is 0.5. We iterate over all detected objects to find the best match. An object should be present in the majority of the images to be included as a final mask. Next, we check the first augmented image for any remaining unused objects (a possible scenario when an object is not detected in the original image but is detected in any of the augmented ones), and look for matching unassigned objects on other augmentations. Next, we check the second augmented image for detected objects, and perform the same operations. We repeat this process until the majority voting criterion can be theoretically satisfied (in half of the images at a maximum). An average binary object mask is created by majority pixel voting on paired objects.

For U-Net the merging process is straightforward as it is not instance aware, so we simply sum and average all the dis-augmented probability maps. It yields a floating point image that needs to be converted to a binary mask. A simple element-wise thresholding at the value of 0.5 converts the soft masks into binary masks (Fig. 1, right).

### Test-time augmentation evaluation

We have evaluated the test-time augmentation model on our test dataset predictions (see the previous section for details) compared to ground truth masks using the following evaluation strategies.

In case of Mask R-CNN we used the same metric as at the Data Science Bowl 2018. It calculates the mean average precision (mAP) at different intersection over union (IoU) thresholds. The thresholds (t) are in the range of [0.5, 0.95] with a step of 0.05. An object is considered true positive when the IoU with ground truth is greater than the threshold, false positive when the predicted object has no associated ground truth object or the overlap is smaller than the threshold, and false negative when the ground truth object has no associated predicted object.

$$IoU(A,B)=\frac{|A\cap B|}{|A\cup B|}$$

Thus, mAP for an image is calculated as follows:

$$\frac{1}{|thresholds|}={\sum }_{t}\,\frac{TP(t)}{TP(t)+FP(t)+FN(t)}$$

Next, we calculate the average for all images in the test set. The final score is a value between 0 and 1.

U-Net predictions were evaluated using the intersection over union metric, executed at the pixel level. We summed up the prediction and ground truth binary masks, then we simply counted the pixels that are greater than one (i.e. the intersection), and divided the resulting values with the number of pixels greater than zero. The resulting value is a score ranging from 0 to 1.

As described above, we have evaluated the predictions with applying TTA (*merged*) and without applying TTA (*original*). Next, we have evaluated TTA’s performance by calculating the difference as *delta* = *merged* − *original*.

## Results

We have evaluated the performance of TTA on two datasets, named ‘Fluorescent’ (fluorescent microscopy images) and ‘Tissue’ (histopathology images) datasets, described in the “Dataset acquisition and description” section in detail. Each of them was split in 3 different ways to have approximately 5% (one holdout set), 15% (cross-validation, 6 splits for each) and 30% (one holdout set) as a test set. By using such versatile data collected from different sources and representing a wide variety of experimental conditions, as well as by the test set splits, we aimed to present the truly general performance of TTA, and demonstrate how robustly it works. Regarding that most of these images were used in a Data Science competition, and some additional images came from other sources, our final datasets are similar to real-world scenarios.

Our choice of the two popular deep learning architectures, Mask R-CNN (yielding instances) and U-Net (semantic segmentation) also served the purpose of testing robustness, as the tasks of semantic and instance segmentation are different, and require different approaches to apply the same method to them. For each dataset/split, we have trained separate U-Net and Mask R-CNN models. Then, we have evaluated the performance of TTA for each model’s checkpoint (checkpoints were made for each epoch of training: in case of U-Net, a total of ‘15’ sets, i.e. every 10th epoch was designated as a checkpoint for cross-validation splits 2–6) as described in the “Test-time augmentation evaluation” subsection. Next, we performed statistical tests to assess whether the improvement of the performance is significant.

**Figure 3:**
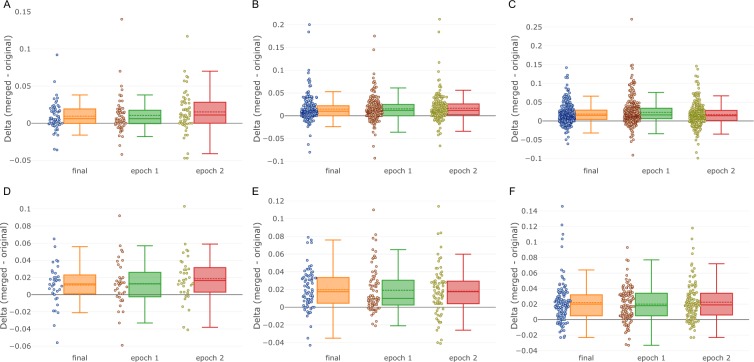
TTA performance for Mask R-CNN. TTA performance (*delta* = *merged* − *original*). Each point represents an image. Dashed line - mean, solid line - median. (**A**) Fluorescent set 5. (**B**) Fluorescent set 15 (cross-validation split 1). (**C**) Fluorescent set 30. (**D**) Tissue set 5. (**E**) Tissue set 15 (cross-validation split 1). (**F**) Tissue set 30. Orange boxplot - the final model (epoch 3), green boxplot - model trained for 1 epoch, red boxplot - model trained for 2 epochs.

In the case of Mask R-CNN, TTA on average has provided an improved performance for all dataset splits and for all model checkpoints. The average mAP score *delta* is about 0.01 for all “Fluorescent” and “Tissue_5” sets and 0.02 for the other sets. In all scenarios, TTA has improved the score for most of the images (see Fig. 3 and Supplementary Fig. 1 for cross-validation splits 2–6). Such a *delta* value usually corresponds for better segmentation borders and a reduced rate of false positive or/and false negative detections.

**Figure 4:**
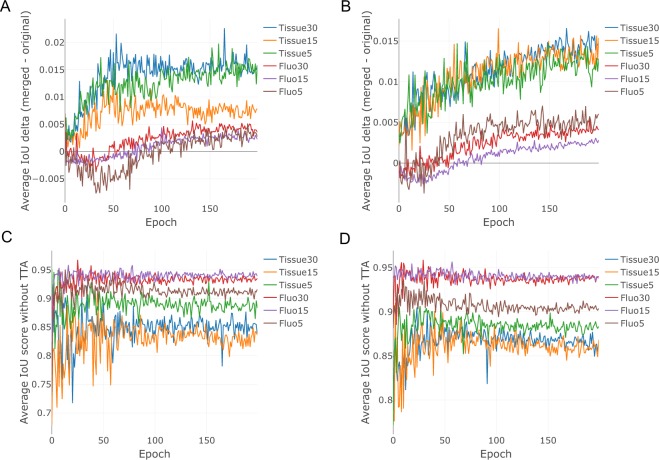
Average performance of TTA for U-Net with different training and test augmentations. (**A**) Average TTA performance trained without augmentations over epochs. (**B**) Average TTA performance trained with augmentations over epochs. (**C**) Average performance without TTA without augmentations during training. (**D**) Average performance without TTA with augmentations during training. Tissue15 and Fluorescent15 stand for the first cross-validation split.

In the case of U-Net, we have evaluated the performance at each epoch during training. For the “Tissue” dataset TTA has demonstrated a performance gain for all epochs. In case of the “Fluorescent” dataset, a slight decline in the performance of TTA was observed during early (first 30–50) epochs, which has turned positive after further training (Fig. 4A,B). After about epoch 50, the performance without TTA was seen to fluctuate without a clear trend in all cases (Fig. 4C,D), while the performance with TTA tended to rise for almost all cases, except in the case of the “Tissue” dataset, where no augmentations were used for training (Fig. 4A). A slight decline or a slight improvement in the score is usually related to cell borders (as the most uncertain regions in the images). In some cases, TTA helps to eliminate artifacts and rarely occurring false positive/false negative objects.

For some images TTA has significantly improved the final prediction. Examples of such cases for both U-Net and Mask R-CNN are shown in Fig. 2.

We have performed Wilcoxon paired test for each dataset/split/checkpoint for the Mask R-CNN results. P-values in all cases have passed the threshold value of 0.05. For U-Net, the test was performed on the means of each 10th epoch (20 vs 20 data points) for each dataset/split. The P-values are shown in Supplementary Table 4.

**Figure 5:**
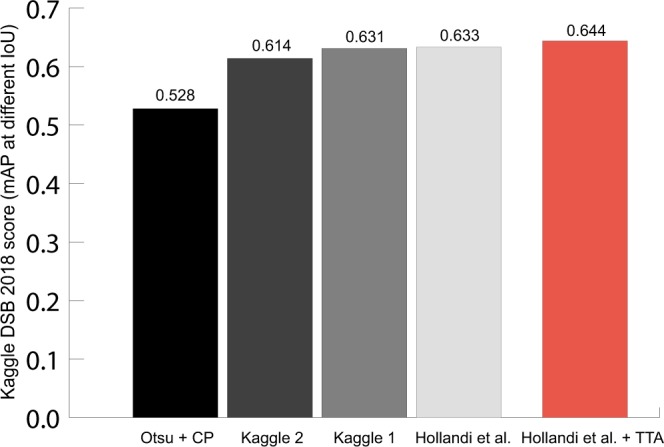
DSB Stage 2 scores for various methods (CellProfiler, Kaggle DSB 2018 2nd and 1st places, Hollandi *et al*.^5^ method and the same method with TTA). The red bar shows the highest score.

Applying TTA on the DSB2018 (stage2) test set of images has improved performance significantly, surpassing the best performing method^5^ by 0.011 in the DSB scoring metric, which is identical to the mAP used in this paper and the output of which was a set of instance segmented masks (Fig. 5). In the context of data science competitions, when the scores are rather dense, we consider this improvement as significant (difference between 2nd and 1st place on DSB 2018 was only 0.017).

The results without TTA and *delta* values for each set are available as Supplementary Materials (Supplementary Table 1. U-Net when augmentations during training were used, Supplementary Table 2. U-Net when augmentations during training were not used, Supplementary Table 3. Mask R-CNN when augmentations during training were used).

## Conclusions

We have performed experiments to estimate test-time augmentation’s performance for two popular deep learning frameworks trained to segment nuclei in microscopy images. Our results indicate that on average TTA can provide higher segmentation accuracy compared to predicting based on the original images only, even though for some images the results might be marginally worse.

TTA mostly affects the objects’ borders, but in uncertain cases it can help to fit whole objects (remove false positives or add true positives, especially in case of Mask R-CNN). Overall, in most cases, TTA improves segmentation accuracy. The main use case of TTA is the analysis of uncertain regions in segmentation. However, the high cost of TTA, related to the fact that multiple times more predictions are required for the same object, is also an issue to be considered. Therefore, TTA is mainly recommended for use when the basic cost of prediction is low.

## Supplementary information

Supplementary Figure 1.

Supplementary Table 1.

Supplementary Table 2.

Supplementary Table 3.

Supplementary Table 4.
