# Author Correction: Test-time augmentation for deep learning-based cell segmentation on microscopy images

**DOI:** 10.1038/s41598-021-81801-8

**Published:** 2021-02-02

**Authors:** Nikita Moshkov, Botond Mathe, Attila Kertesz-Farkas, Reka Hollandi, Peter Horvath

**Affiliations:** 1 Biological Research Centre, Szeged, Hungary; 2 University of Szeged, Szeged, Hungary; 3 National Research University, Higher School of Economics, Moscow, Russia; 4 Institute for Molecular Medicine Finland, University of Helsinki, Helsinki, Finland

Correction to: *Scientific Reports* 10.1038/s41598-020-61808-3, published online 19 March 2020

This Article contains errors in Figure 4, where segmentation score (Jaccard index) results for U-Net experiments and scores of both cases, with test-time augmentation and without test-time augmentation, are incorrect. The correct Figure 4 appears below as Figure 1.

**Figure 1:**
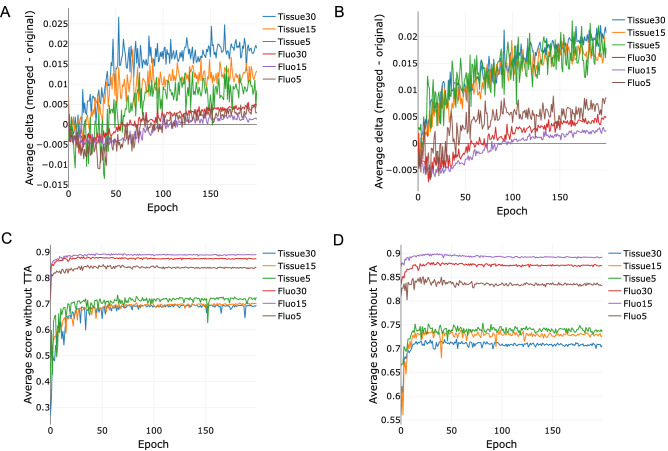
A correct version of the original Figure 4.

Consequently, in the Results section,

“For the “Tissue” dataset TTA has demonstrated a performance gain for all epochs. In case of the “Fluorescent” dataset, a slight decline in the performance of TTA was observed during early (first 30–50) epochs, which has turned positive after further training (Figure 4 A,B).”

should read:

“For the “Tissue” dataset TTA has demonstrated a performance gain for almost all epochs. In case of the “Fluorescent” dataset, a slight decline in the performance of TTA was observed during early (first 50–100) epochs, which has turned positive after further training (Figure 4 A,B).”

Finally, resulting from the incorrect segmentation score (Jaccard index) results for U-Net experiments, Supplementary Tables 1, 2 and 4 published with this Article contain errors.

The corrected Supplementary Information files are linked to this correction notice.

## Supplementary information

Supplementary Table 1.

Supplementary Table 2.

Supplementary Table 4.

