# Supplementary material for: Test-time augmentation for deep learning-based cell segmentation on microscopy images
Source: Sci Rep. 2020 Mar 19;10:5068. doi: 10.1038/s41598-020-61808-3 (PMC7081314; doi:10.1038/s41598-020-61808-3)
Supplement: Supplementary file 1 — Supplementary Figure 1. [file 41598_2020_61808_MOESM1_ESM.pdf]

Nikita Moshkov, Botond Mathe, Attila Kertesz-Farkas, Reka Hollandi, Peter Horvath

Test-time augmentation for deep learning-based cell segmentation on microscopy images

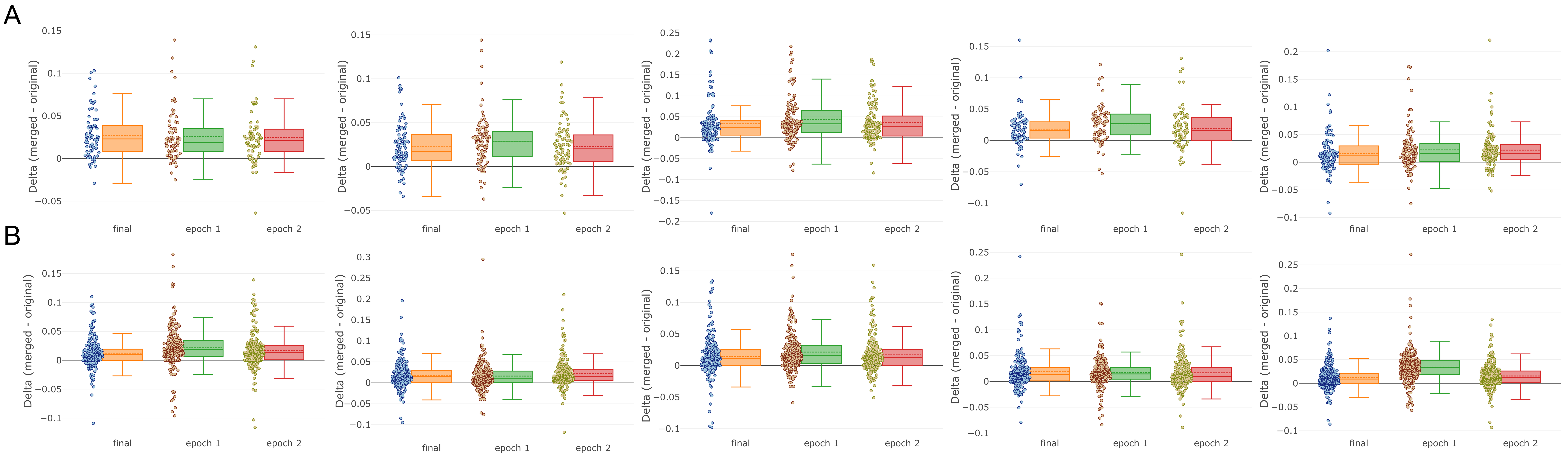

Supplementary Figure 1. TTA performance for Mask R-CNN

TTA performance (delta = merged-original). Each point represents an image. Dashed line - mean, solid line - median. A | Tissue 15 set cross-validation folds 2-6 (left to right) B | Fluorescent 15 set cross-validation folds 2-6 (left to right)

Orange boxplot - the final model (epoch 3), green boxplot - model trained for 1 epoch, red boxplot - model trained for 2 epochs.
